# A nationwide study of multidrug-resistant tuberculosis in Portugal 2014–2017 using epidemiological and molecular clustering analyses

**DOI:** 10.1186/s12879-019-4189-7

**Published:** 2019-07-01

**Authors:** Olena Oliveira, Rita Gaio, Carlos Carvalho, Margarida Correia-Neves, Raquel Duarte, Teresa Rito

**Affiliations:** 1 Population Health Research Domain, Life and Health Sciences Research Institute (ICVS), School of Medicine, University of Minho, Gualtar Campus, 4710-057 Braga, Portugal; 2 ICVS/3B, PT Government Associate Laboratory, 4710-057 Braga, 4805-017 Guimarães, Portugal; 3 EPIUnit, Instituto de Saúde Pública, Universidade do Porto, 4050-600 Porto, Portugal; 4 Department of Mathematics, Faculty of Sciences, Porto, Portugal; 5 Centre of Mathematics, University of Porto, Porto, Portugal; 6 Department of Public Health, Northern Regional Health Administration, 4000-078 Porto, Portugal; 7 Multidisciplinary Unit for Biomedical Research (UMIB), Institute of Biomedical Sciences Abel Salazar, University of Porto, 4050-013 Porto, Portugal; 8 Departamento de Ciências da Saúde Pública e Forenses e Educação Médica, Faculdade de Medicina, Universidade do Porto, 4200-319 Porto, Portugal; 9 Pulmonology Department, Centro Hospitalar de Vila Nova de Gaia/Espinho EPE, 4400-129 Vila Nova de Gaia, Portugal

**Keywords:** Multidrug-resistant tuberculosis, Epidemiology, Transmission, Risk factor

## Abstract

**Background:**

Increasing multidrug-resistant tuberculosis (MDR-TB) incidence is a major threat against TB eradication worldwide. We aim to conduct a detailed MDR-TB study in Portugal, an European country with endemic TB, combining genetic analysis and epidemiological data, in order to assess the efficiency of public health containment of MRD-TB in the country.

**Methods:**

We used published MIRU-VNTR data, that we reanalysed using a phylogenetic analysis to better describe MDR-TB cases transmission occurring in Portugal from 2014 to 2017, further enriched with epidemiological data of these cases.

**Results:**

We show an MDR-TB transmission scenario, where MDR strains likely arose and are transmitted within local chains. 63% of strains were clustered, suggesting high primary transmission (estimated as 50% using MIRU-VNTR data and 15% considering epidemiological links). These values are higher than those observed across Europe and even for sensitive strains in Portugal using similar methodologies. MDR-TB cases are associated with individuals born in Portugal and evolutionary analysis suggests a local evolution of strains. Consistently the sublineage LAM, the most common in sensitive strains in Europe, is the more frequent in Portugal in contrast with the remaining European MDR-TB picture where immigrant-associated Beijing strains are more common.

**Conclusions:**

Despite efforts to track and contain MDR-TB strains in Portugal, their transmission patterns are still as uncontrolled as that of sensitive strains, stressing the need to reinforce surveillance and containment strategies.

**Electronic supplementary material:**

The online version of this article (10.1186/s12879-019-4189-7) contains supplementary material, which is available to authorized users.

## Background

Tuberculosis (TB) remains a high burden disease worldwide with persistent areas where elimination is still a distant goal. Despite declining incidence of TB in the last decades, multidrug-resistant TB (MDR-TB) poses a major threat for WHO’s 2035 goal of TB elimination [1]. MDR-TB, defined by resistance of *Mycobacterium tuberculosis* (Mtb) to isoniazid and rifampicin (RR), emerges as consequence of ineffective treatment or incompletion or inappropriate following up of the cases, translating into the evolution of resistant strains with consequent increments in patient morbidity and mortality, and further transmission [2].

WHO estimated a worldwide incidence of around 560,000 cases of MDR/RR-TB per year and a rate of 7.4 cases per 100,000 individuals [3]. In Europe, MDR/RR-TB incidence rate was 12.0 per 100,000 individuals, the highest among the regions considered by WHO [3], but results could be biased towards regions with more detailed monitoring programmes on drug-susceptibility testing (DST). MDR-TB notification rate in Portugal has been increasing since 2012 at a rate of 0.8 and 3.7% among new cases and previously treated patients, respectively [4].

TB incidence has been decreasing in Portugal. In 2017, TB notification-rate was 17.8 per 100,000 individuals [5], however 24.8 and 26.0 in the large urban centres of Lisbon and Porto respectively. The proportion of MDR-TB remains at 1% of all TB cases. 80% of MDR-TB patients had no previous TB treatment [5], suggesting mostly primary transmission of MDR strains but without supporting epidemiological studies confirming this scenario.

Previous genetic studies, evaluating clinical isolates collected in Lisbon Health Region, reported high prevalence of MDR-TB with most cases concentrating in two monophyletic clades (Lisbon 3 and Q1) [6, 7]. A database developed by Perdigão and colleagues [8] constitutes the largest collaborative effort to catalogue Mtb diversity in Portuguese-speaking countries. It includes 423 MDR-TB isolates (129 from Portugal) within a larger dataset of 1447 clinical samples, validating Latin-American-Mediterranean (LAM: lineage 4) as the most common sub-lineage in Portugal. These studies are mostly from Lisbon hospitals [6–8], but information on epidemiological characteristics of the cases is lacking, undermining the design of control strategies for preventing and reducing MDR-TB incidence [9].

Although MIRU-VNTR is becoming outdated in the study of TB transmission, mostly due to the overestimation of recent transmission [10–15], it is nevertheless the most used genotyping method implemented across Europe by Public Health authorities in a recent survey [16]. Although conclusions on transmission should be performed with extreme caution, the large body of work accumulated for MIRU-VNTR across Europe and worldwide allows statistics to be methodically compared for regions and scenarios still lacking whole genome data.

In this study we combined an in depth genetic analysis of strain genotyping already published from Portugal [10], with epidemiological data from MDR-TB cases diagnosed between 2014 and 2017, collected as part of the routine functions of Public Health services. We aim to assess the dynamics of MDR-TB emergence and transmission, including the identification of associated risk factors, and last to establish the rate of probable recent transmissions against newly developed resistant strains. These statistics will be compared with results for sensitive strains in Portugal and other MDR-TB transmission scenarios in other developed countries using similar approaches in order to assess the relative efficiency of public health measures for containing MDR-TB in Portugal.

## Methods

### Data collection

We identified and extracted data from all culture-confirmed MDR-TB cases, diagnosed between 2014 and 2017, from the national TB Surveillance System (SVIG-TB), containing epidemiological, clinical and laboratory data. Epidemiological information collected includes gender, age at time of TB diagnosis, country of origin, place of residence (parish), presence or absence of alcohol or drug misuse, HIV status, description of previous TB treatment and clinical characteristics of TB presentation (site of disease). Information about previous contact of patients with other TB cases was provided by Public Health services, using as linking variables date of birth, sex and place of residence, in order to identify possible epidemiological links between MDR-TB cases.

Mycobacterial interspersed repetitive unit-variable-number tandem repeat (MIRU-VNTR) genotypes for sensitive and MDR strains were collected from genotypic works that characterised strains isolated from Portugal, namely [7, 17, 18]. Linked epidemiological and MIRU-VNTR data was only possible to MDR-TB cases described between 2014 and 2017.

### Ethical approval

This work was carried out in accordance with the recommendations by the Ethics Sub-commission of Life and Health Sciences from the University of Minho (SECVS 135/2015), by the Ethics Committee for Health of Lisbon and Tagus Valley Region Health Administration (9854/CES/2018) and the Ethics Committee for Health of the Northern Region Health Administration (ARSN 127/2018). All procedures were in accordance with the ethical standards of the responsible committees and with the Helsinki Declaration, as revised in 2008.

### Cluster analysis

Clustering analysis was performed as described before in [19]: a cluster was defined as two or more cases with the same MIRU-VNTR profiles. The proportion of recent transmission was calculated by the “n minus one” method [20], using number of cases clustered – number of clusters/number of cases with a strain type.

An epidemiological link was defined between two cases when cases had identified others as contacts, or when cases shared a family or household connection. “Possible” epidemiological links were defined as cases from the same geographical area, with common social or behavioural traits (e.g. workplace, drug use). The number of epidemiological links was used to recalculate an adjusted proportion of recent transmission taking into consideration only those clustered cases further supported by an epidemiological link.

### Evolutionary analysis of MDR-TB strains

The phylogenetic reconstruction of MIRU-VNTR profiles was done using median networks [21]. We used two algorithms consecutively implemented in the network software (fluxus engineering), the reduced median followed by the median joining algorithm, as previously described in [18]. We used this hybrid approach weighting the 24 loci according to their allelic diversity [22].

Isolates that present similar MIRU genotypes were referred as genotypically clustered, representing potential episodes of direct TB transmission. Networks were used to construct the most parsimonious trees. Cladograms were visualized in Figtree v.1.3.3 (http://tree.bio.ed.ac.uk/software/figtree/). The classification of the strains in lineages was done using miruvntrplus (http://www.miru-vntrplus.org).

### Statistical analysis

Data were summarized by descriptive statistics (absolute and relative frequencies or median and range) according to the nature of the variables. Chi-square or Fisher’s exact tests were used to evaluate the independence between two categorical variables, while Mann–Whitney U-test compared the distributions of two independent continuous variables. We identified risk factors associated with MDR-TB transmission, comparing patient’s socio-demographic and clinical characteristics associated with clustering, that were investigated using logistic regression (comparing cases with unique genotypes against those within clusters). Statistical analyses were performed with SPSS version 18.0 (PASW Statistics 18), and *p*-values below 0.05 were considered statistically significant.

## Results

### MDR-TB epidemiology

**Fig. 1:**
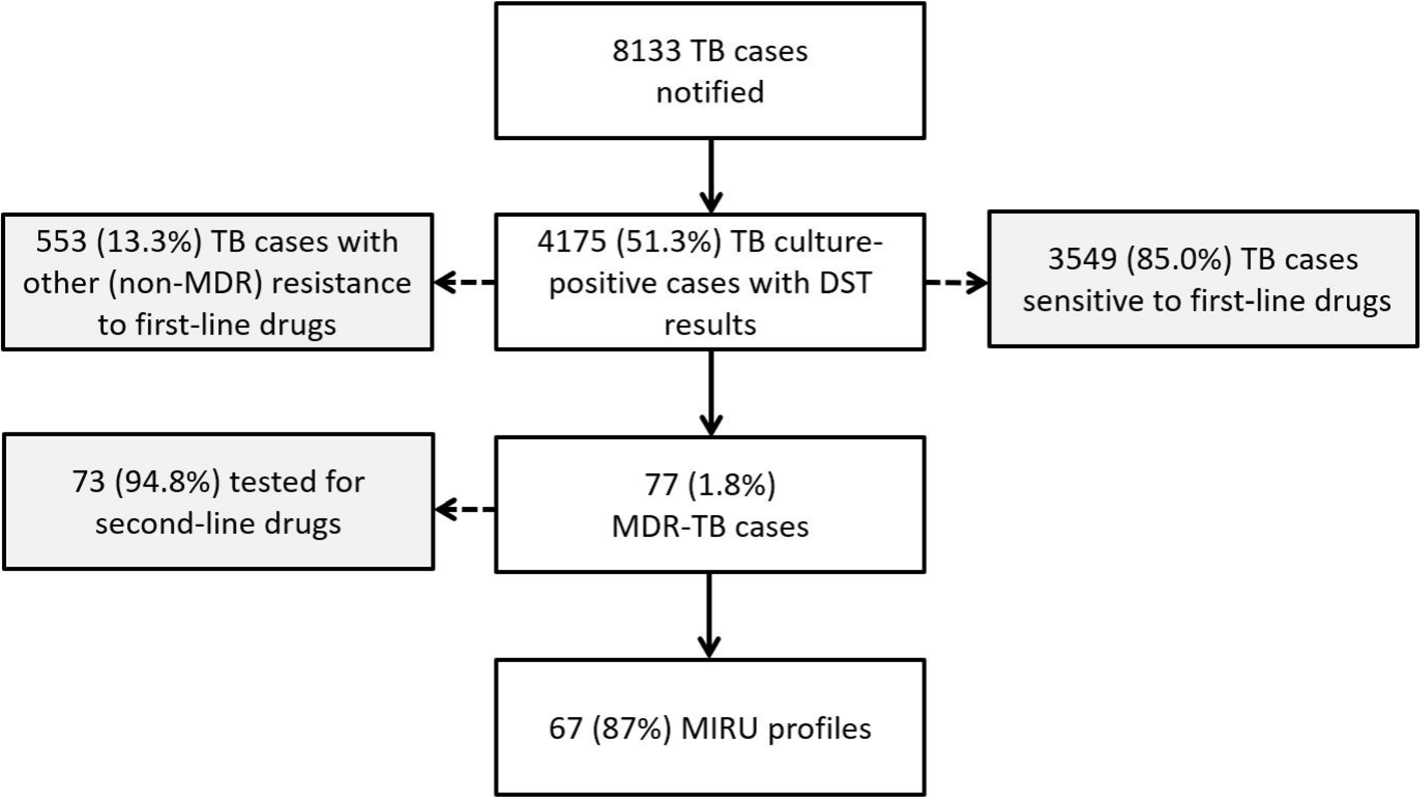
Tuberculosis cases notified and proportion of multidrug-resistant tuberculosis cases identified in Portugal between 2014 and 2017

In Portugal, from January 2014 until December 2017, 8133 TB cases were notified, of which 4175 (51.3%) cases were culture-confirmed and had results of first-line DST (Fig. 1), identifying 77 MDR-TB cases (~ 1% of total cases).

**Fig. 2:**
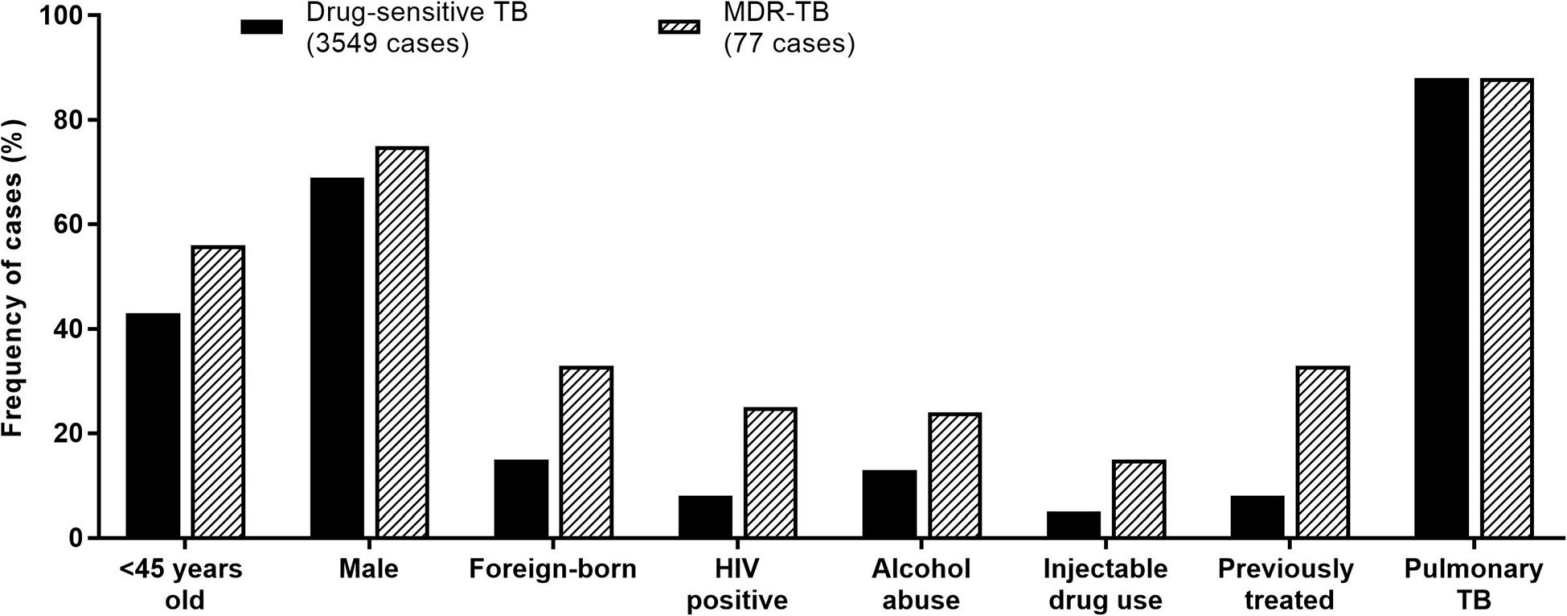
Differences in prevalence of socio-demographic and clinical characteristics between patients with drug-sensitive tuberculosis (*n* = 3549) and with multidrug-resistant tuberculosis (*n* = 77). All the cases where diagnosed between 2014 and 2017

We compared patients with drug-sensitive TB and MDR-TB in the same period (Fig. 2, Additional file 2: Table S1). Patients with MDR-TB were younger: the median age was 43 years (range 20–75) and 55.8% were below 45 years. MDR-TB patients presented higher frequencies of foreign-born individuals (32.9% vs. 15.3%), HIV infected (24.7% vs. 8.0%), alcohol abusers (23.9% vs. 13.2%), injectable drugs users (14.7% vs. 4.6%) and with previous TB treatment (32.9% vs. 7.5%). Among the foreign-born patients, 11 (44%) cases entered the country in the two previous years.

### Cluster analysis and potential transmission links

**Fig. 3:**
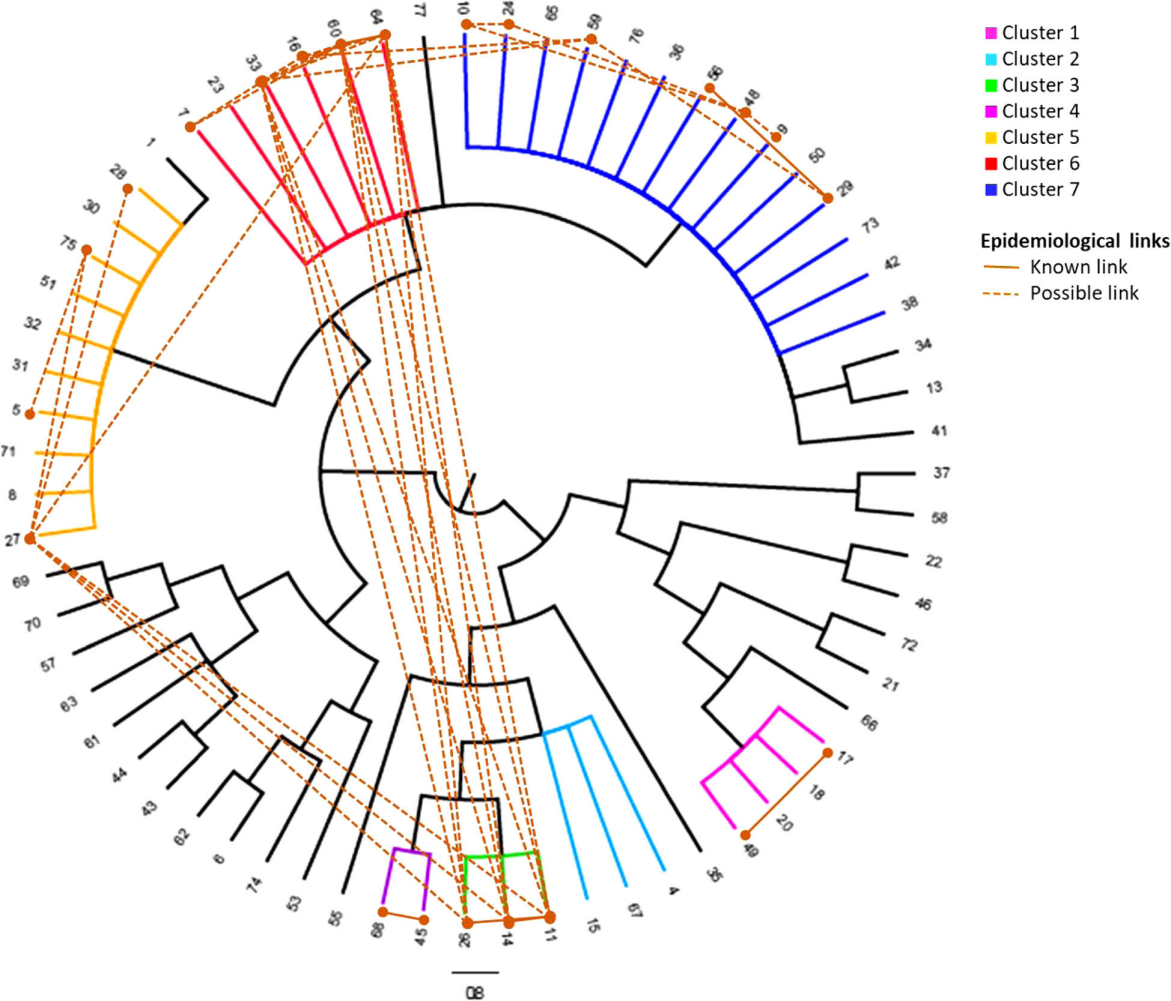
Cladogram representing the phylogenetic tree obtained with MIRU-VNTR profiles from 67 cases of multidrug-resistant tuberculosis and epidemiological links identified between cases. Clusters 1 to 7 are coloured as follow: pink (cluster 1), light blue (cluster 2), green (cluster 3), purple (cluster 4), orange (cluster 5), red (cluster 6) and dark blue (cluster 7)

Among 67 MDR-TB cases, 46 cases (69%) were LAM, eight (12%) cases were sublineage Haarlem, two (3%) URAL strains and one (1%) were sublineage X-type. Ten (15%) isolates belonged to Lineage 2, sublineage Beijing. 42 cases were identified in seven MIRU-VNTR clusters ranging from 2 to 14 cases (Table 1; Fig. 3). These MIRU-VNTR clusters showed good correspondence to previously defined clusters on the whole genome level [23] and each MIRU-defined cluster has a set of defined specific mutations in drug-resistance associated genes attesting for their significance as clades. The three largest clusters [5–7], and contained 10, 6 and 14 cases, respectively, with most of these corresponding to Portugal-born individuals, with pulmonary disease, diagnosed in the LTV region. Twenty-five cases presented unique strains, possible outside introductions or newly developed MDR-TB strains (Additional file 3: Table S2). Genetically, the proportion of cases attributable to recent transmission was 52.2%. While this value is strongly overestimated, as MIRU clusters can correspond to clades dating to several years, it allows the identification of circulating clades of MDR strains in the Portuguese scenario.

**Table 1:** Characteristics of MIRU-VNTR clusters, included MIRU profile, identified *Mycobacterium tuberculosis* sub-lineage, patients’ country of origin, residence area in Portugal (including North, Central region, Lisbon and Tagus Valley, South and autonomous islands Madeira and Azores), patients’ risk factors (identified at diagnosis, namely alcohol abuse, drug misuse, residence in shelters or community residence and history of previous tuberculosis treatment)

Cluster	MIRU-24 loci profile	*M.tuberculosis* sublineage	*n*	Country of origin	Residence area in Portugal	Risk factors	Disease site	Previous TB treatment
1	243,244,331,234 424,153,334,332	Haarlem	4	Portugal	North	1 alcohol abuse	3 pulmonary, 1 extra-pulmonary	No
2	244,233,352,644 425,153,353,823	Beijing	3	Guinea-Bissau	2 LVT, 1 Central	No	1 pulmonary, 1 extra-pulmonary, 1 unknown	1 no, 1 yes, 1 unknown
3	244,233,352,644 425,173,343,723	Beijing	3	Portugal	LVT	Injectable drugs use	Pulmonary	No
4	244,233,352,644 425,183,353,823	Beijing	2	Portugal	1 Madeira, 1 Central	No	Pulmonary	No
5	244,213,132,324 114,142,532,822	LAM	10	7 Portugal, 2 Cape Verde, 1 Mozambique	LVT	3 alcohol abuse, 2 injectable drug use, 1 shelter	7 pulmonary, 2 extra-pulmonary, 1 unknown	5 no, 4 yes, 1 unknown
6	244,213,232,324 116,143,532,822	LAM	6	5 Portugal, 1 Cape Verde	5 LVT, 1 Central	3 alcohol abuse, 2 injectable drug use	Pulmonary	4 no, 2 yes
7	244,213,232,424 116,143,532,822	LAM	14	12 Portugal, 2 Angola	9 LVT, 2 Central, 3 North	4 alcohol abuse, 4 injectable drug use	10 pulmonary, 3 extra-pulmonary, 1 unknown	6 no, 7 yes, 1 unknown

To corroborate the established genetic connections, we analysed information provided by the Public Health services, identifying 17 links (5 known, 12 possible) between cases within five clusters (Fig. 3):

- In cluster 1, containing four cases from the Northern region, two patients lived in the same parish;
- The three patients within cluster 3 were drug-users, attended the same community care facilities and two were close relatives;
- Patients of cluster 4 were friends;
- Two cases of the cluster 6 were relatives; also users of the community care facilities mentioned above for cluster 3;
- Two cases of the cluster 7 were close relatives. One was a civil construction worker with possible contact with two patients of the cluster, also construction workers in the same area.

The proportion of cases attributable to recent transmission after adjustment for detected epidemiological links was 14.9%.

### Risk factors associated with clustering

To identify particular risk factors underlying active transmission of MDR-TB, we compared clustered cases (likely recent transmission cases) against unique cases. In the univariate analysis, being born in Portugal (OR 3.67; 95% CI 1.24–10.88; *p* = 0.019) and alcohol abuse (OR 10.15; 95% CI 1.22–84.39; *p* = 0.032) were associated with clustering (Table 2). Nevertheless, the table shows that alcohol abuse is a good identifier for clustered cases and its low frequency within the unique cases turns the logistic regression model impractical. After adjustment for gender and age, being Portugal-born was the only independently clustering-associated variable (adjusted OR 3.64; 95% CI 1.16–11.47; *p* = 0.027).

**Table 2:** Assessment of patient’s characteristics and risk factors that could be associated with multidrug-resistant *Mycobacterium tuberculosis* clustering of cases, considering the cases reported between 2014 and 2017

MDR-TB patient Characteristic		Unknown	Unique (*n* = 25)	Clustered (*n* = 42)	Univariate analysis	
			n (%)	n (%)	OR (95% CI)	*p*-value
Age group	< 45 years old	0	16 (64.0)	21 (50.0)	Ref	0.267
	≥45 years old		9 (36.0)	21 (50.0)	1.78 (0.64–6.91)	
Gender	Female	0	7 (28.0)	9 (21.4)	Ref	0.543
	Male		18 (72.0)	33 (78.6)	1.43 (0.46–4.47)	
Country of origin	Foreign-born	1	12 (50.0)	9 (21.4)	Ref	**0.019**
	Native		12 (50.0)	33 (78.6)	**3.67 (1.24–10.88)**	
HIV status	Negative	4	21 (87.5)	27 (69.2)	Ref	0.109
	Positive		3 (12.5)	12 (30.8)	3.11 (0.78–12.46)	
Alcohol abuse	No	6	22 (95.7)	26 (68.4)	Ref	**0.032**
	Yes		1 (4.3)	12 (31.6)	**10.15 (1.22–84.39)**	
Injectable drug use	No	8	20 (87.0)	29 (80.6)	Ref	0.525
	Yes		3 (13.0)	7 (19.4)	1.61 (0.37–6.98)	
TB treatment history	Never treated	4	15 (62.5)	25 (64.1)	Ref	0.898
	Previously treated		9 (37.5)	14 (35.9)	0.93 (0.33–2.68)	
Site of disease	Pulmonary	4	24 (42.9)	32 (57.1)	n/a	n/a
	Extra-pulmonary		0 (0.0)	7 (100.0)		

*CI* confidence interval, *OR* odds ratio, *Ref* reference

Statistically significant values are indicated in bold

### Genetic contextualization of Portuguese MDR-TB strains

Aiming at a clearer picture of emergence and spread of Mtb strains, we collected and combined published Portuguese MIRU-VNTR data [7, 10, 18].

**Fig. 4:**
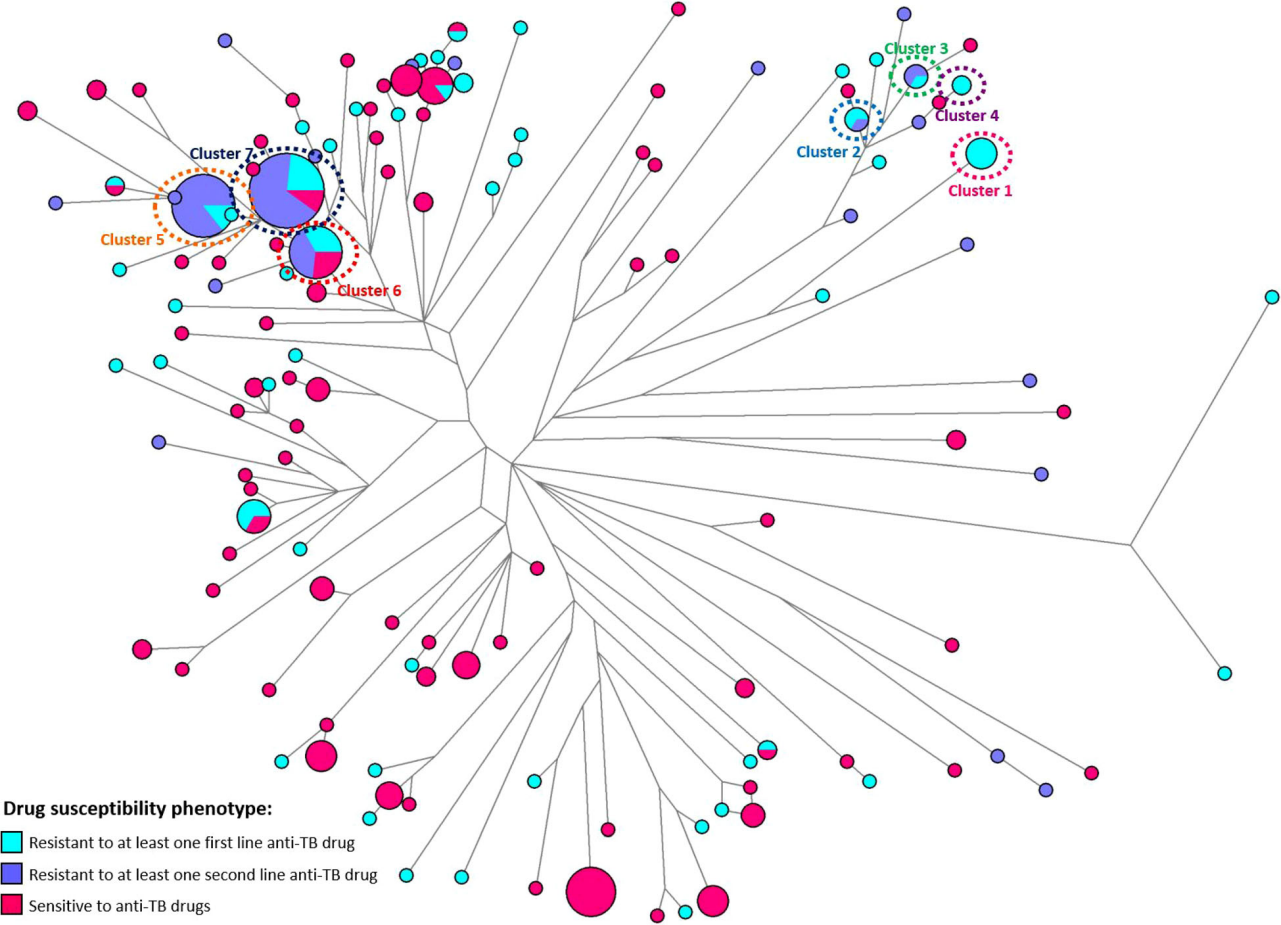
Reduced median network of *Mycobacterium tuberculosis* genotypes, representing the current scenario of multidrug-resistant tuberculosis in Portugal and how these strains are phylogenetically related with the sensitive tuberculosis strains. The network displays 67 profiles of multidrug-resistance strains [17], 144 strains from the Northern region [18] and 56 strains with different levels of resistance [7]. Clusters detected in previous analyses and reported throughout the paper are highlighted

The evolutionary networks are shown in Fig. 4 and Additional file 1: Figure S1 Networks represent genotypes (circles), where branch length is proportional to genetic distance. Size of the circles is proportional to frequency of cases with the same genotype, being hypothetical clusters of transmission. MDR clusters (Table 1) are highlighted in the figures. Genotypes from strains resistant to at least one second-line anti-TB drug often appear within clusters (Fig. 4) but with individual strains scattered across the network, similarly to sensitive and first-line resistant strains. One aspect to explore with caution, given the low resolution of MIRU-VNTR markers, is that deeper clusters often include sensitive, first-line resistant and MDR strains within the Portuguese population (being cluster 1 the exception). A common ancestry of MDR and sensitive strains in the same area suggests that MDR strains are likely the result of evolution of strains occurring within local chains of transmissions in Portugal and not brought from abroad, often without time for differentiation between both in MIRU markers.

From the network analysis, MDR-TB cluster distribution is similar to the pattern observed with drug-sensitive strains, as described, for example, for Porto Urban area [18], with the existence of several prominent clusters in both suggesting active transmission, or at least the maintenance of several circulating MDR-TB clades within the population. Moreover, general clustering statistics are very similar to that dataset, corresponding to 63% of the MDR-TB cases being clustered, against 59.7% in the drug-sensitive TB cases within Porto (*p* = 0.883, not significant). Considering both datasets, the percentage of cases attributed to recent transmission based on MIRU-VNTR data is 52.2% against 43.8%, higher in MDR cases. While the statistics are overestimated using MIRU-VNTR they are directly comparable and both suggest the circulation of specific Mtb clades within the population in terms of sensitive and MDR strains.

For refining the geography of MDR-TB, we inspected the origin of the drug-resistance cases. The main clusters described in Lisbon [7], are also present in the Centre and Northern regions of the country. Also, while many of the single genotypes are present in immigrants (Additional file 1: Figure S1) possible recent introductions, clustered cases, often clustering with sensitive (Fig. 4), are mostly present in autochthonous individuals, reinforcing a possible evolution and transmission of MDR strains within indigenous transmission chains [18].

### History of previous TB treatment

Twenty-three (34%) of the 67 MDR-TB cases with MIRU-VNTR profiles reported previous TB treatment, lacking DST and genotypic data on these past infections.

One individual reported three previous TB treatments. This individual was Portuguese, inmate and injectable drug user. He perished during the fourth treatment with a strain resistant to all first-line drugs and 6 s-line drugs tested, appearing isolated in the genetic networks, pinpointing this case expectedly as one of emergence of resistance. Six other cases reported two previous treatment courses but display no specific evolutionary pattern, being either clustered or isolated.

Within the 23 MDR-TB cases that reported a previous TB treatment, nine had an unique genotype, appearing isolated in the network, possibly representing independent evolution of MDR strains. In opposite, 14 genotypes were in clusters, likely transmission events. The frequency of individuals with previous TB treatment in clustered genotypes is basically the same as the frequency in non-clustered individuals, 36 and 38%, respectively (Table 2). This might reflect the fact that risk of reinfection and inadequate treatment are likely associated with the same risk groups, making the individuals likely vessels for either acquiring new strains or development of MDR.

## Discussion

There are various initiatives worldwide aiming to contain and minimise the impact of MDR-TB. Nevertheless, an integrative scenario of MDR-TB transmission dynamics in Portugal is virtually inexistent. During a 4-year period (2014–2017) MDR-TB patients were younger, with higher prevalence of foreign-born individuals, HIV infected, alcohol abusers, injectable drug users and with previous TB treatment, when compared with drug-sensitive patients. Previous TB treatment is known as a strong risk factor for MDR-TB [24–27], and young age, HIV infection, being foreign-born and frequent consumption of alcohol have also been reported in several countries associated with development of MDR-TB [24, 25, 27, 28]. Several of these risk factors are underlying ineffective completion of previous treatments.

We identified seven MIRU-VNTR clusters, where the three largest clusters belonged to sublineage LAM, mostly corresponding to Portugal-born individuals, with pulmonary disease, diagnosed in the LTV region. Being Portugal-born was the independent risk factor statistically associated with MDR-TB recent transmission, suggesting that contrarily to the scenario in other developed countries MDR-TB is not strongly associated with foreign individuals. This trend is also supported by the frequency of the main genotyped lineages.

According to the European Centre for Disease Control (ECDC) surveillance report, Beijing was the most common genotypic lineage among MDR-TB strains isolated in 2015 in Europe (60.0%), followed by LAM (17.6%) [29], despite LAM being more prevalent in sensitive strains (as in Ireland [30] and Belgium [31], for example). By contrast, here, the most common genotypic lineage in MDR-TB was LAM (69%), followed by Beijing (15%) and Haarlem (12%), similarly to a study on sensitive strain in Northern Portugal (LAM represented 61.1%) [18]. The similar prevalence of LAM in sensitive and MDR-strains strengthens the hypothesis that MDR-TB in Portugal is evolving within autochthonous chains of transmission and being transmitted similarly to drug-sensitive TB.

Clusters 5, 6 and 7 correspond to previously identified MDR-TB clusters Q1, Lisboa3-A and Lisboa3-B in the LTV region [6, 7], reflecting continuous circulation of these strains in the region extending to other regions of Portugal and abroad (Q1 and Lisboa3-B were included in cross-border clusters reported by ECDC, being present in the UK and France [29]). While we suggested an origin of these clusters within the autochthonous transmission chains, determining exact source and direction of transmission between countries would require higher discriminatory power and deeper epidemiological investigations [32].

The proportion of clustered cases in our study (63%) was generally higher than most studies using similar methodologies, for example in the UK, Switzerland and USA [19, 33, 34] but similar to a Portuguese sensitive sample (59.7%) following the same criteria [18]. We estimated recent transmission to 52.2% using MIRU-VNTR data and 14.9 after adjustment for epidemiological data. While MIRU-VNTR data largely overestimates recent transmission estimates [16, 35], the epidemiological data likely underestimates direct transmission given the difficulty in assessing all relevant epidemiological information using only conventional contact tracing data in complex transmission chains [18]. Nevertheless, these 14.9% were still twice as high as what was reported in UK [19] and Switzerland [34] using similar approaches. The higher prevalence of MDR-TB (above 50%) displaying no previous treatment provides evidence for high rate of primary transmission of MDR-TB in Portugal, also supported by a higher estimated rate of transmission in MDR than sensitive strains in Portugal, against the expected European trend. Independently of the clustering being overestimated, in the sense that MIRU clusters could date up to decades in some instances, it reflects the existence of circulating genetic clades being maintained in the population.

## Conclusion

This study has limitations in terms of analysed period and genotyping, but it offers nevertheless a strong effort to assess MDR-TB scenario in Portugal using a more detailed combined MIRU-VNTR and epidemiological analysis. While the recent transmission rates are widely overestimated by the application of MIRU-VNTR data, there is nevertheless a striking scenario that relates to a high percentage of circulating strains from the same clades (when compared with sensitive strains and MDR-TB in other countries) than what could be expected given the public health efforts to contain MDR-TB. Taking into account the possible scenarios of emergence of MDR-TB, it is necessary to readjust measures to decrease transmission, including for example, improvement of earlier diagnosis and better adherence to treatment in specific regions of the country, including the support of community institutions focusing on specific population groups, and a faster and integrative genotyping protocol for early identification of clustered cases.

## Additional files

Additional file 1: **Figure S1.** Reduced median network, representing the current scenario of multidrug-resistant TB in Portugal and how these strains are grouped together with the sensitive TB strains. The network displays 67 profiles of *Mycobacterium tuberculosis* strains [17], 144 strains from the Northern region [18] and 56 strains with different levels of resistance [7]. The migratory status of the cases is highlighted: green cases are natives from Portugal, yellow are foreigners and grey are individuals that on diagnosis (or publication) did not disclosure migratory status. Clusters detected in previous analyses and reported throughout the paper are highlighted. (DOCX 572 kb)

Additional file 2: **Table S1.** Assessment of patient’s characteristics of drug-sensitive tuberculosis and multidrug-resistant tuberculosis, considering the cases reported between 2014 and 2017. (DOCX 20 kb)

Additional file 3: **Table S2.** Characteristics of non-clustered cases, including the ID displayed in Fig. 3, identified *Mycobacterium tuberculosis* lineage and sub-lineage, patients’ country of origin, residence area in Portugal (including North, Central region, Lisbon and Tagus Valley, South and autonomous islands Madeira and Azores), patients’ risk factors (identified at diagnosis, namely alcohol abuse, drug misuse, residence in shelters or community residence and history of previous TB episodes and consequent treatment). (DOCX 20 kb)

## Data Availability

The genetic datasets analysed during the current study are available within the manuscripts referenced by us and also in http://cplp-tb.ff.ulisboa.pt/. The raw epidemiological dataset generated and analysed during the current study is not publicly available and it is property of the TB National programme. All the relevant information analysed is contained within this manuscript.

## Abbreviations

SVIG: Surveillance System
TB: Tuberculosis
CI: Confidence interval
DST: Drug susceptibility testing
ECDC: European Centre for Disease Control
HIV: Human immunodeficiency virus
LAM: Latin-American-Mediterranean
LTV: Lisbon and Tagus Valley
MDR-TB: Multidrug-resistant tuberculosis
MIRU-VNTR: Mycobacterial interspersed repetitive units–variable number tandem repeat
Mtb: *Mycobacterium tuberculosis*
OR: Odds ratio
PASW: Predictive Analytics Software
RR: Rifampicin-resistant
SPSS: Statistical Package for the Social Sciences
TB: Tuberculosis
WHO: World Health Organization
