# Supplementary material for: A nationwide study of multidrug-resistant tuberculosis in Portugal 2014–2017 using epidemiological and molecular clustering analyses
Source: BMC Infect Dis. 2019 Jul 1;19:567. doi: 10.1186/s12879-019-4189-7 (PMC6604307; doi:10.1186/s12879-019-4189-7)
Supplement: Supplementary file 1 — Figure S1. Reduced median network, representing the current scenario of multidrug-resistant TB in Portugal and how these strains are grouped together with the sensitive TB strains. The network displays 67 profiles of Mycobacterium tuberculosis strains [17], 144 strains from the Northern region [18] and 56 strains with different levels of resistance [7]. The migratory status of the cases is highlighted: green cases are natives from Portugal, yellow are foreigners and grey are individuals that on diagnosis (or publication) did not disclosure migratory status. Clusters detected in previous analyses and reported throughout the paper are highlighted. (DOCX 572 kb) [file 12879_2019_4189_MOESM1_ESM.docx]

**Additional File 1**


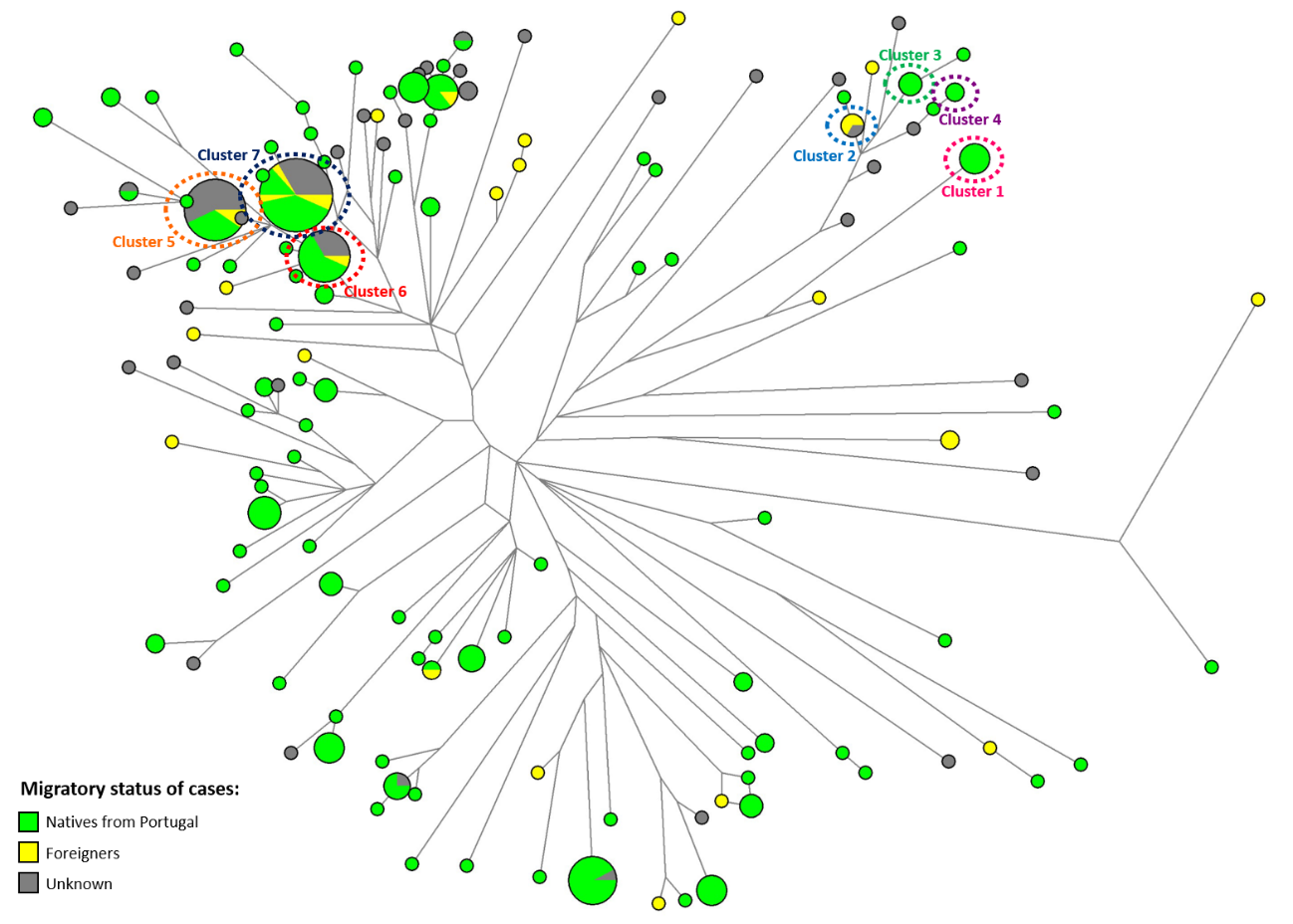


**Figure-S1. Reduced median network, representing the current scenario of multidrug-resistant TB in Portugal and how these strains are grouped together with the sensitive TB strains. The network displays 67 profiles of *Mycobacterium tuberculosis* strains (10), 144 strains from the Northern region (12) and 56 strains with different levels of resistance (7). The migratory status of the cases is highlighted: green cases are natives from Portugal, yellow are foreigners and grey are individuals that on diagnosis (or publication) did not disclosure migratory status. Clusters detected in previous analyses and reported throughout the paper are highlighted.**
