# Supplementary material for: A nationwide study of multidrug-resistant tuberculosis in Portugal 2014–2017 using epidemiological and molecular clustering analyses
Source: BMC Infect Dis. 2019 Jul 1;19:567. doi: 10.1186/s12879-019-4189-7 (PMC6604307; doi:10.1186/s12879-019-4189-7)
Supplement: Supplementary file 2 — Table S1. Assessment of patient’s characteristics of drug-sensitive tuberculosis and multidrug-resistant tuberculosis, considering the cases reported between 2014 and 2017. (DOCX 20 kb) [file 12879_2019_4189_MOESM2_ESM.docx]

**Additional File 2**

**Table-S1. Assessment of patient’s characteristics of drug-sensitive tuberculosis and multidrug-resistant tuberculosis, considering the cases reported between 2014 and 2017.**

| **Patients characteristics** | | **Unknown** | **Total** | **Drug-sensitive TB** | **MDR-TB** | **p-value** |
| --- | --- | --- | --- | --- | --- | --- |
|  |  |  |  | nº (%) | nº (%) |  |
| Age, years | Median  (min, max) | 0 | 3626 | 48 (0-96) | 43 (20-75) | 0.111 |
| Age group | <45 years old  ≥45 years old | 0 | 3626 | 1538 (43.3)  2011(56.7) | 43(55.8)  34(44.2) | **0.038** |
| Gender | Female | 0 | 3626 | 1098(30.9) | 19(24.7) | 0.292 |
|  | Male |  |  | 2451(69.1) | 58(75.3) |  |
| Country of origin | Foreign-born | 1 | 3625 | 543(15.3) | 25(32.9) | **<0.001** |
|  | Native |  |  | 3006(84.7) | 51(67.1) |  |
| HIV status | Negative | 4 | 3622 | 3265(92.0) | 55(75.3) | **<0.001** |
|  | Positive |  |  | 284(8.0) | 18(24.7) |  |
| Alcohol abuse | No | 218 | 3408 | 2898(86.8) | 54(76.1) | **0.014** |
|  | Yes |  |  | 439(13.2) | 17(23.9) |  |
| Injectable drug use | No | 223 | 3403 | 3183(95.4) | 58(85.3) | **<0.001** |
|  | Yes |  |  | 152(4.6) | 10(14.7) |  |
| TB treatment history | Never treated | 4 | 3622 | 3283(92.5) | 49(67.1) | **<0.001** |
|  | Previously treated |  |  | 266(7.5) | 24(32.9) |  |
| Site of disease | Pulmonary | 4 | 3622 | 3113(87.7) | 64(87.7) | 1.000 |
|  | Extra-pulmonary |  |  | 436(12.3) | 9(12.3) |  |
