# Supplementary material for: A nationwide study of multidrug-resistant tuberculosis in Portugal 2014–2017 using epidemiological and molecular clustering analyses
Source: BMC Infect Dis. 2019 Jul 1;19:567. doi: 10.1186/s12879-019-4189-7 (PMC6604307; doi:10.1186/s12879-019-4189-7)
Supplement: Supplementary file 3 — Table S2. Characteristics of non-clustered cases, including the ID displayed in Fig. 3, identified Mycobacterium tuberculosis lineage and sub-lineage, patients’ country of origin, residence area in Portugal (including North, Central region, Lisbon and Tagus Valley, South and autonomous islands Madeira and Azores), patients’ risk factors (identified at diagnosis, namely alcohol abuse, drug misuse, residence in shelters or community residence and history of previous TB episodes and consequent treatment). (DOCX 20 kb) [file 12879_2019_4189_MOESM3_ESM.docx]

**Additional File 3**

**Table-S2. Characteristics of non-clustered cases, including the ID displayed in Figure-3, identified *Mycobacterium tuberculosis* lineage and sub-lineage, patients’ country of origin, residence area in Portugal (including North, Central region, Lisbon and Tagus Valley, South and autonomous islands Madeira and Azores), patients’ risk factors (identified at diagnosis, namely alcohol abuse, drug misuse, residence in shelters or community residence and history of previous TB episodes and consequent treatment).**

| **ID**  **Figure-3** | **Lineage** | **Sub-lineage** | **Country of origin** | **Residence area in Portugal** | **Risk factos** | **Site of disease** | **TB treatment history** |
| --- | --- | --- | --- | --- | --- | --- | --- |
| 1 | 4 | LAM | Portugal | LTV | Drug use, Prison | Pulmonary | Yes |
| 6 | 4 | LAM | Belarus | LTV | No | Pulmonary | No |
| 13 | 4 | LAM | Portugal | LTV | Alcohol abuse | Pulmonary | No |
| 21 | 4 | Haarlem | Portugal | North | Drug use, Homeless | Pulmonary | No |
| 22 | 4 | X | Andorra | North | No | Pulmonary | Yes |
| 41 | 4 | LAM | Guinea | LTV | Community residence | Pulmonary | Yes |
| 34 | 4 | LAM | Portugal | LTV | No | Pulmonary | No |
| 37 | 4 | URAL | Ukraine | LTV | No | Pulmonary | No |
| 35 | 2 | Beijing | Portugal | LTV | Drug use, prison | Pulmonary | Yes |
| 43 | 4 | LAM | Portugal | North | No | Pulmonary | Yes |
| 44 | 4 | LAM | Portugal | North | No | Pulmonary | No |
| 46 | 4 | Haarlem | Moldova | Algarve | No | Pulmonary | No |
| 57 | 4 | LAM | Angola | LTV | No | Pulmonary | Yes |
| 61 | 4 | LAM | Angola | LTV | No | Pulmonary | No |
| 55 | 2 | Beijing | Ukraine | LTV | Community residence | Pulmonary | Yes |
| 62 | 4 | LAM | Portugal | Central | No | Pulmonary | Yes |
| 53 | 4 | LAM | Portugal | Central | No | Pulmonary | No |
| 66 | 4 | Haarlem | Angola | Central | No | Pulmonary | No |
| 69 | 4 | LAM | Angola | LTV | No | Pulmonary | No |
| 77 | 4 | LAM | Portugal | LTV | No | Pulmonary | No |
| 72 | 4 | Haarlem | Portugal | North | No | Pulmonary | No |
| 70 | 4 | LAM | Angola | North | No | Pulmonary | No |
| 58 | 4 | URAL | Portugal | Central | No | Pulmonary | No |
| 74 | 4 | LAM | Angola | Central | No | Pulmonary | Yes |
| 63 | 4 | LAM | Unknown | LTV | Unknown | Unknown | Unknown |
